# Resistant cumin cultivar, GC-4 counters *Fusarium oxysporum* f. sp. *cumini* infection through up-regulation of steroid biosynthesis, limonene and pinene degradation and butanoate metabolism pathways

**DOI:** 10.3389/fpls.2023.1204828

**Published:** 2023-10-17

**Authors:** Darshan T. Dharajiya, Nitin Shukla, Maharshi Pandya, Madhvi Joshi, Amrutlal K. Patel, Chaitanya G. Joshi

**Affiliations:** Gujarat Biotechnology Research Centre (GBRC), Department of Science and Technology, Government of Gujarat, Gandhinagar, Gujarat, India

**Keywords:** *Cuminum cyminum* L., fusarium wilt, disease resistance, induced systemic resistance, transcriptome, *Fusarium oxysporum* f. sp. *cumini*

## Abstract

Cumin (*Cuminum cyminum* L.), an important spice crop belonging to the Apiaceae family is infected by *Fusarium oxysporum* f. sp. *cumini* (*Foc*) to cause wilt disease, one of the most devastating diseases of cumin adversely affects its production. As immune responses of cumin plants against the infection of *Foc* are not well studied, this research aimed to identify the genes and pathways involved in responses of cumin (cv. GC-2, GC-3, GC-4, and GC-5) to the wilt pathogen. Differential gene expression analysis revealed a total of 2048, 1576, 1987, and 1174 differentially expressed genes (DEGs) in GC-2, GC-3, GC-4, and GC-5, respectively. In the resistant cultivar GC-4 (resistant against *Foc*), several important transcripts were identified. These included receptors, transcription factors, reactive oxygen species (ROS) generating and scavenging enzymes, non-enzymatic compounds, calcium ion (Ca^2+^) transporters and receptors, R-proteins, and PR-proteins. The expression of these genes is believed to play crucial roles in conferring resistance against *Foc*. Gene ontology (GO) analysis of the up-regulated DEGs showed significant enrichment of 19, 91, 227, and 55 biological processes in GC-2, GC-3, GC-4, and GC-5, respectively. Notably, the resistant cultivar GC-4 exhibited enrichment in key GO terms such as ‘secondary metabolic process’, ‘response to reactive oxygen species’, ‘phenylpropanoid metabolic process’, and ‘hormone-mediated signaling pathway’. Furthermore, the Kyoto Encyclopedia of Genes and Genomes (KEGG) pathway analysis revealed the enrichment of 28, 57, 65, and 30 pathways in GC-2, GC-3, GC-4, and GC-5, respectively, focusing on the up-regulated DEGs. The cultivar GC-4 showed enrichment in pathways related to steroid biosynthesis, starch and sucrose metabolism, fatty acid biosynthesis, butanoate metabolism, limonene and pinene degradation, and carotenoid biosynthesis. The activation or up-regulation of various genes and pathways associated with stress resistance demonstrated that the resistant cultivar GC-4 displayed enhanced defense mechanisms against *Foc*. These findings provide valuable insights into the defense responses of cumin that could contribute to the development of cumin cultivars with improved resistance against *Foc*.

## 1 Introduction

Cumin (*Cuminum cyminum* L.) from the Apiaceae family is a small annual and herbaceous plant (Mnif and Aifa, 2015). Cumin seeds have several applications in industries like spice, pharmaceutical, and food, and are also used in traditional Ayurvedic medicine to treat several diseases (Arun et al., 2016; Sharma et al., 2016; Srinivasan, 2018; Choudhary et al., 2021). India is the leading country in the world in cumin cultivation area, production, consumption, and supplier/exporter since 2010 (Meena et al., 2018; Kumar et al., 2021). In India, Gujarat has been the leading state of cumin production and productivity followed by Rajasthan (Kumar et al., 2021). However, cumin production is adversely affected by biotic factors, mainly fungal diseases such as wilt (*Fusarium oxysporum* f. sp. *cumini* (*Foc*)), blight (*Alternaria burnsii*), and powdery mildew (*Erysiphe polygoni*) (Dange, 1995). Among the fungal diseases, wilt, caused by *F. oxysporum* f. sp. *cumini*, is considered a severe disease that hinders worldwide cumin production. It causes high yield losses, up to 100% in severely damaged areas with disease-promoting environments (Abo-Elyousr et al., 2022). Because of the prolonged existence and accumulation of *Foc* in soil, there is no effective control strategy available at present. Therefore, understanding of *Foc* resistance mechanism, effective chemical-free and environmentally-safe strategies to reduce the substantial loss caused by *Foc* in cumin production are important and highly required.

The wilt pathogen can infect plants in all the stages of growth, but the severity of wilt increases with the age of the plant. In the severe infection, the plants and leaves wilt and die. Seeds formed by infected plants are thin, small, and shriveled. The seeds are often contaminated during harvesting and the pathogen spreads to new areas (Lodha and Mawar, 2014). Wilt disease in cumin can be managed by cultural, biological, and chemical approaches. While various fungicides may provide effective control of *Foc*, the excessive use of these chemicals poses significant hazards to the environment and human health, as well as the issue of resistance development (Abo-Elyousr et al., 2022). With the absence of an effective wilt management approach, research efforts have primarily focused on identifying resistant sources and utilizing them in resistance breeding programs. There are limited resistant sources against wilt pathogens in the available germplasm throughout the world (Lodha and Mawar, 2014).

In recent years, transcriptome analysis in different crops like flax (Boba et al., 2021), cotton (Hou et al., 2021), banana (Dong et al., 2020), mung bean (Chang et al., 2021), and common bean (Leitão et al., 2021) have been performed to elucidate the genes and pathways for fusarium wilt (caused by different subspecies or special forms of *F. oxysporum* in different crops) resistance. Response of plants towards *F. oxysporum* infection varied in different crops. The defense responses of cumin to *Foc* infection are not well known even though this information is very important to plan effective approaches against destructive diseases. The identification of genes responsible for resistance against *Foc* can be of great importance for understanding the detailed molecular mechanism of resistance in cumin to fusarium wilt disease and ultimately utilizing the genes related to *Foc* resistance for cultivar improvement in cumin. Hence, the main objective of this study was to reveal the genes and pathways responsible for *Foc* resistance in cumin cultivars by transcriptome analysis and to understand the mechanism of resistance.

## 2 Materials and methods

### 2.1 Plant sample collection

Plant samples of four cumin cultivars namely, Gujarat cumin-2 (GC-2) (susceptible to wilt), GC-3 (resistant to wilt), GC-4 (resistant to wilt), and GC-5 (moderately resistant to wilt) (Meena et al., 2010) were collected from Seed Spices Research Station, Sardarkrushinagar Dantiwada Agricultural University (SDAU), Jagudan (23.5177° N Latitude, 72.4125° E Longitude), Mehsana, Gujarat in the year of 2020. The samples of healthy and wilt diseased plants of all the cultivars were collected at their reproductive stage from the control plot and wilt sick plot, respectively. The plants showing wilt disease symptoms were collected and used for the isolation of pathogens. The pure culture of isolated fungus was observed under microscope and identified by PCR amplification using ITS1 (5’-TCCGTAGGTGAACCTGCGG-3’) and ITS4 (5’-TCCTCCGCTTATTGATATGC-3’) primers and sequencing of ITS region of ribosomal DNA by automatic capillary sequencer ABI 3500XL (Applied Biosystems, CA, USA). The sequence data were used for BLAST analysis on NCBI.

### 2.2 RNA extraction, library preparation, and transcriptome sequencing

Plant samples (aerial parts of the plant) were powdered using liquid nitrogen in a mortar and pestle. Total RNA was extracted using RNeasy® Plant Mini Kit (Qiagen, Germany) according to the manufacturer’s instructions. Extracted RNA samples were checked on denatured agarose gel. The purity of RNA samples was checked using a QIAxpert spectrophotometer (Qiagen, Germany). The quantity and integrity of the RNA were assessed by the Qubit™ 4 Fluorometer (Thermo Fisher Scientific, MA, USA) and Agilent Bioanalyzer 2100 system (Agilent Technologies, CA, USA), respectively. A total of eight cDNA libraries (GC-2-H, GC-2-I, GC-3-H, GC-3-I, GC-4-H, GC-4-I, GC-5-H, and GC-5-I) from RNA samples were prepared using Ion Total RNA-Seq Kit v2 (Thermo Fisher Scientific, MA, USA). Template preparation was done by Ion Chef™ Instrument (Thermo Fisher Scientific, MA, USA) and sequencing was performed on Ion GeneStudio™ S5 Plus System (Thermo Fisher Scientific, MA, USA). The raw sequences were submitted to sequence read archive (SRA) – NCBI (**Supplementary Table S1**).

### 2.3 Transcript assembly and differential gene expression analysis

The quality assessment of reads was performed by FastQC (v.0.11.9). After a quality check of reads, PRINSEQ++ (v.1.2.4) was used to remove low-quality sequences (Q value <20 and read length <100 bp). The high-quality reads were reconstructed and assembled into unigenes by Trinity (v.2.8.5) with default parameters. The assembled sequences were annotated with Transdecoder (v.5.5.0) for prediction of the open reading frame. The individual samples were mapped and quantified against the assembled transcriptome using salmon (v.1.9.0). The gene expression levels of individual transcripts were estimated by transcripts per million (TPM). The counts data was simulated using the Seqgendiff package (v.1.2.3) (Gerard, 2020) and analysis of differentially expressed genes (DEGs) between infected and healthy plants of each cultivar was performed with the DeSeq2 package (v.1.34.0) in R. The genes with threshold p-value ≤ 0.05 | log2FoldChange (log2FC) ≥ 2 and ≤ −2 were considered significant DEGs. The Venn diagrams for DEGs were drawn by using InteractiVenn (Heberle et al., 2015), an online tool.

### 2.4 Gene ontology (GO) and Kyoto encyclopedia of genes and genomes (KEGG) pathway enrichment analysis

The transcripts from individual samples were annotated using the KOBAS-i (v.3.0) tool (Bu et al., 2021). The biological significance of up- and down-regulated genes was assessed by the KEGG pathway and GO enrichment which provided three ontologies, viz., biological process, cellular component, and molecular function using ShinyGO (v.0.76.3) (Ge et al., 2020). The enrichment analysis was performed by hypergeometric distribution followed by false discovery rate (FDR) correction. The data of annotated DEGs were used in R Studio for graphical visualization with the ggplot2 package (3.4.0) and pheatmap package (1.0.12) from the bioconductor. The illustration for the proposed defense response in cumin against *Foc* was prepared by Microsoft PowerPoint 2016.

## 3 Results

### 3.1 RNA-seq data processing and *de novo* assembly

To study the variation in gene expression in GC-2, GC-3, GC-4, and GC-5, healthy and infected plants of these cultivars were collected. The fungal culture isolated from infected plants was confirmed as *F. oxysporum* by microscopic observation and sequence analysis (**Supplementary File 1**). Transcriptomes of healthy and infected plants of cumin cultivars were analyzed. *De novo* assembly of cumin was constructed using Trinity and individual transcriptome samples were mapped against the *de novo* assembly of cumin. The assembly contained a median contig length of 344 bp with a total assembled 29,836,958 bases. It represented 62,845 ‘genes’ and 65,389 transcripts with GC content of 44.35% and N50 value of 501 bp (**Supplementary Table S2**). A total of 169,992,277 reads were generated from eight libraries with a mean of 21,249,035 reads (**Supplementary Table S3**). The mapping of eight samples with the *de novo* assembly of cumin ranged from 82.19% to 95.42% with an average of 92.2% (**Supplementary Table S3**).

### 3.2 Identification of differentially expressed genes (DEGs)

**Figure 1:**
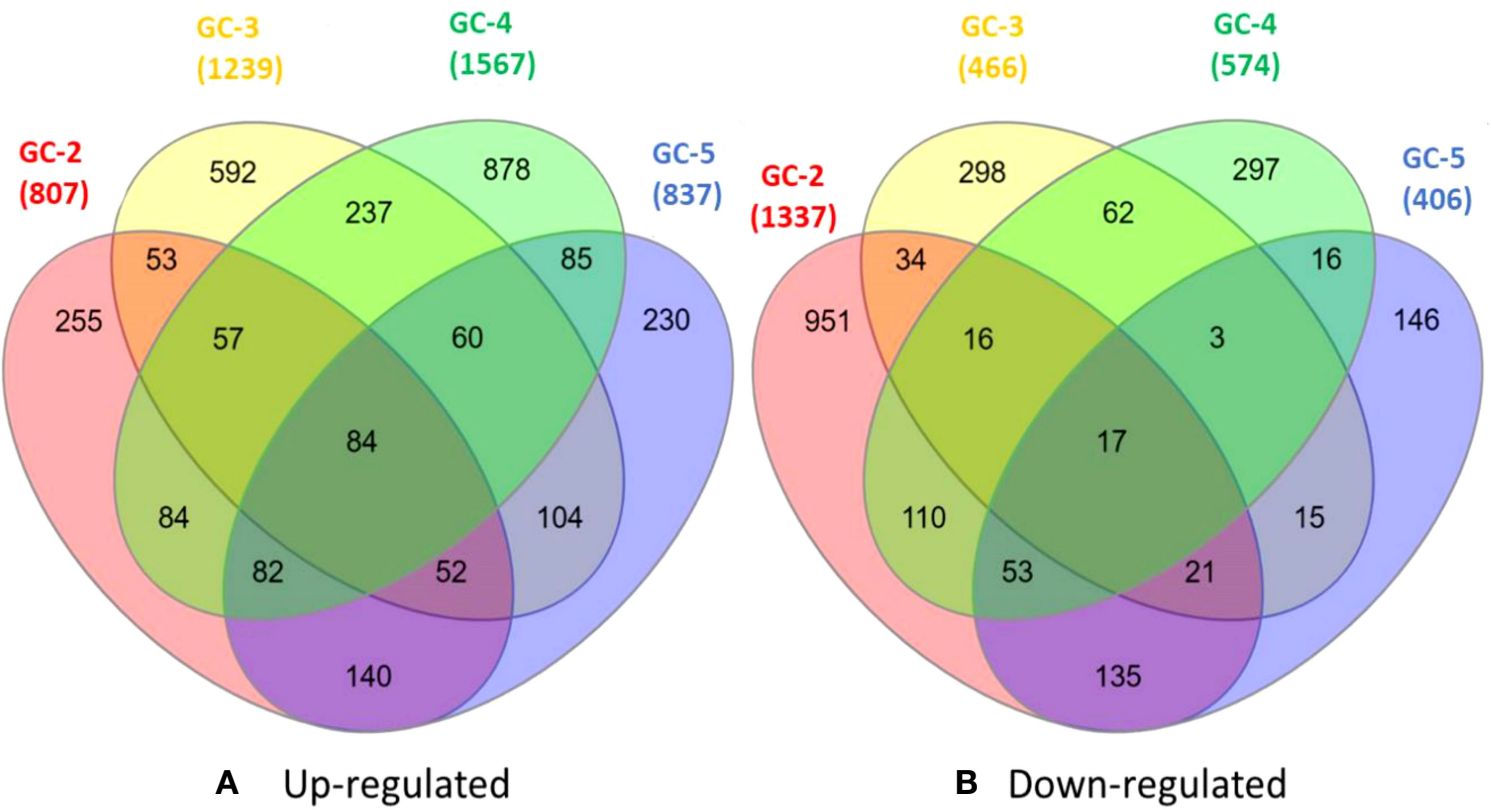
Venn diagrams of DEGs **(A)** up-regulated and **(B)** down-regulated healthy and diseased plants of cumin cultivars (GC-2, GC-3, GC-4 and GC-5). The cut-off set for DEGs was, log2FC ≥ 2 for up-regulated and ≤ −2 for down-regulated. The cut-off for p-value ≤ 0.05 was set for all DEGs.

The healthy and infected samples of each cultivar were used to identify DEGs. The total DEGs were identified with a threshold of p-value ≤ 0.05 and with log2FC ≥ 2.0 and ≤ −2.0 for up- and down-regulated DEGs, respectively. Among all the annotated DEGs, duplicates were removed for further analysis. Among these DEGs, 807, 1239, 1567, and 837 were up-regulated and 1337, 466, 574, and 406 were down-regulated in GC-2, GC-3, GC-4, and GC-5, respectively (**Figure 1**).

**Figure 2:**
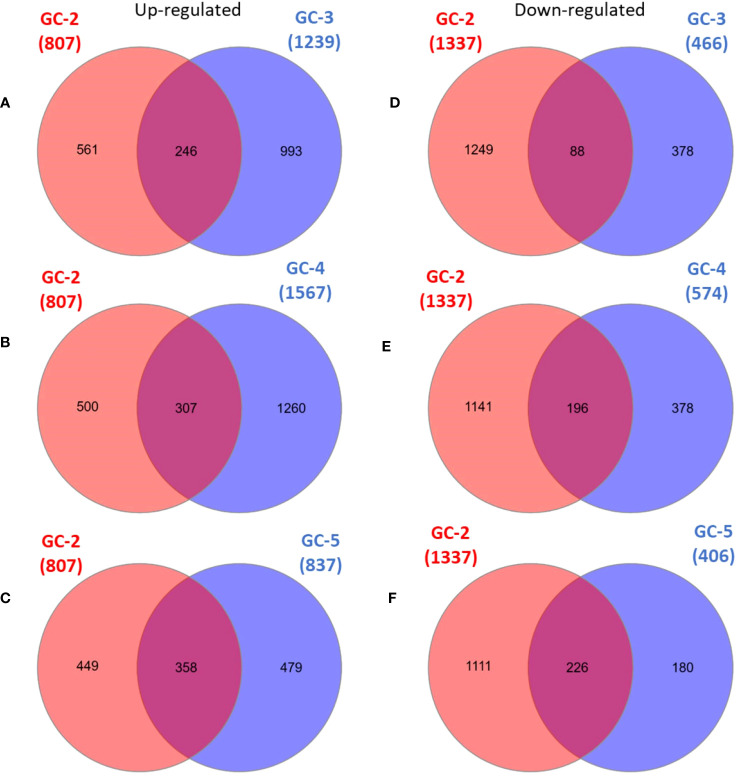
Venn diagrams of DEGs in susceptible cultivar (GC-2) vs resistant (GC-3 and GC-4)/moderately resistant cultivar (GC-5). **(A)** up-regulated DEGs: GC-2 vs GC-3, **(B)** up-regulated DEGs: GC-2 vs GC-4, **(C)** up-regulated DEGs: GC-2 vs GC-5, **(D)** down-regulated DEGs: GC-2 vs GC-3, **(E)** down-regulated DEGs: GC-2 vs GC-4, **(F)** down-regulated DEGs: GC-2 vs GC-5. The cut-off for the log2FC was set ≥ 2 for up-regulated DEGs and ≤ −2 for down-regulated DEGs. The cut-off for p-value was ≤ 0.05 for all DEGs.

The list of DEGs of all the cultivars is given in **Supplementary File 2**. Inter-cultivar comparison of up-regulated DEGs of GC-2 (susceptible) and GC-3 (resistant) showed that 561 and 993 were unique in GC-2 and GC-3, respectively while comparing down-regulated DEGs, 1249 and 378 were unique in GC-2 and GC-3, respectively. By comparing up-regulated DEGs of GC-2 (susceptible) and GC-4 (resistant), 500 and 1260 DEGs were unique in GC-2 and GC-4, respectively while comparing down-regulated DEGs, 1141 and 378 were unique in GC-2 and GC-4, respectively. The unique and common DEGs for susceptible and resistant/moderately resistant cultivars have been shown in **Figure 2**.

**Figure 3:**
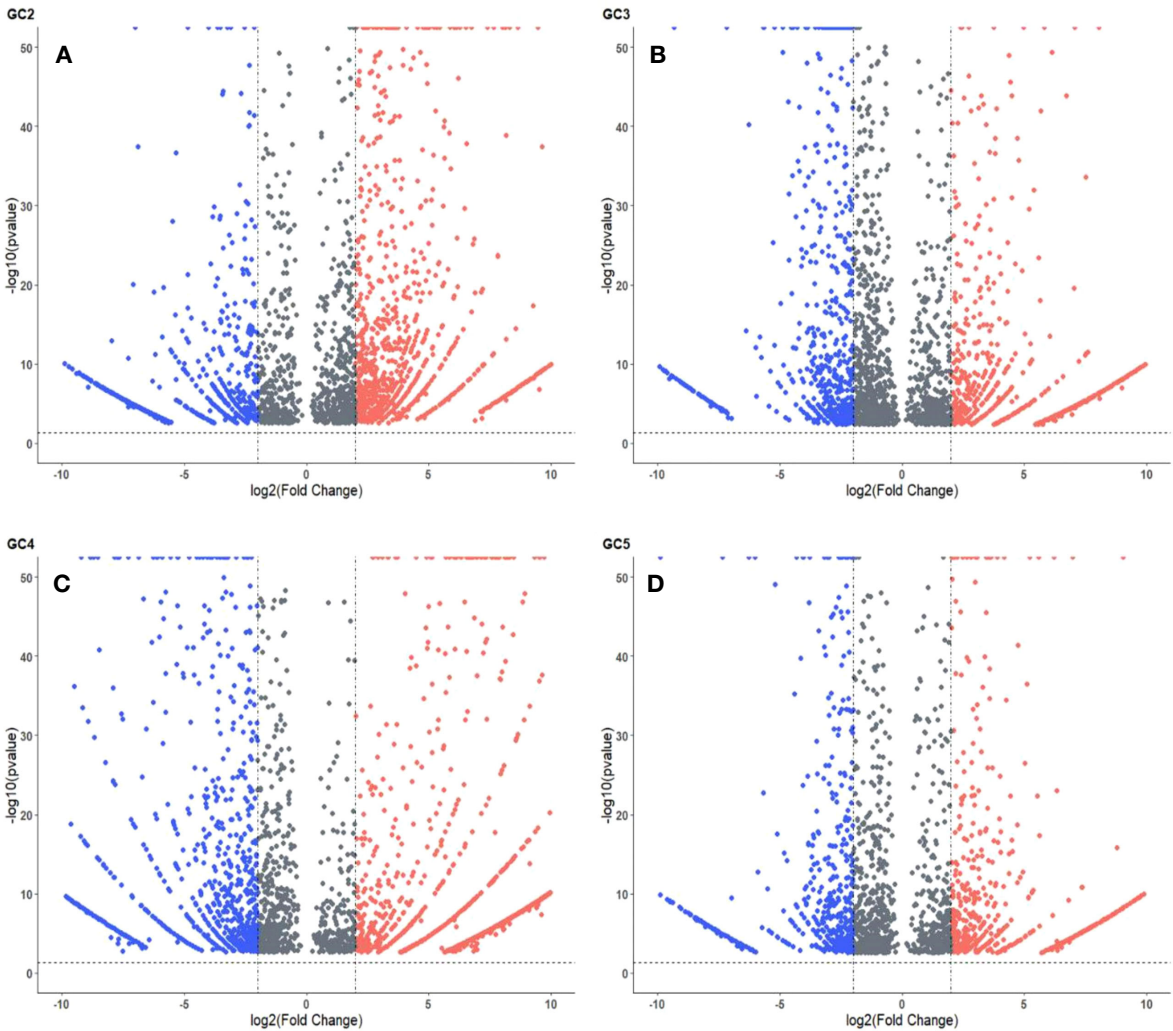
Volcano plot of DEGs in cumin cultivars **(A)** GC2, **(B)** GC3, **(C)** GC4 and **(D)** GC5. The cut-off for the log2FC was set ≥ 2 for up-regulated DEGs and ≤ −2 for down-regulated DEGs. The cut-off for p-value was ≤ 0.05 for all DEGs. Red and blue colors indicate up-regulated and down-regulated DEGs, respectively.

The volcano plots representing the distribution of DEGs in healthy and infected plants of GC-2, GC-3, GC-4, and GC-5 are presented in **Figure 3**. By considering the cut-off p-value ≤ 0.05 and log2FC ≥ 2 for up-regulated DEGs and ≤ −2 for down-regulated DEGs a total of 2048, 1576, 1987, and 1174 DEGs were considered for GC-2, GC-3, GC-4, and GC-5, respectively. More DEGs with high Log2FC were plotted for GC-4 compared to the susceptible cultivar (GC-2). The hierarchical clustering properly divided the healthy plant samples from the infected plant samples representing the differential regulation of genes based on normalized counts for the top fifty DEGs in all four cultivars (**Supplementary Figures S1**–**S4**).

### 3.3 Functional classification of DEGs

**Figure 4:**
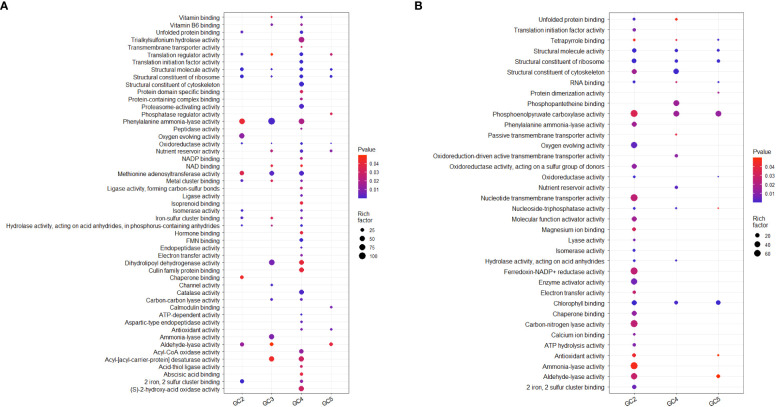
Overview of selected enriched GO terms for molecular function **(A)** up-regulated and **(B)** down-regulated in transcriptome of cumin cultivars in response to *Foc* infection. The color shows p-value (Bejamini Hochberg) and size of the dots represents the rich factor of significant genes involved in each corresponding process. Rich factor is the ratio of the number of DEGs to the total number of genes in a given GO term. In GC-3 cultivar, no significant enrichment was observed for down-regulated DEGs hence not considered in the plot.

Test sets for GO analysis with up-regulated DEGs from all the cultivars resulted in 44, 41, 130, and 22 enriched significant GO terms for molecular function in GC-2, GC-3, GC-4, and GC-5, respectively. The details on no. of genes involved in the pathway and enriched genes in individual pathways in each cultivar are given in **Supplementary File 3**. In GC-4, enriched unique key GO terms for molecular function were ‘transmembrane transporter activity’, ‘structural constituent of cytoskeleton’, ‘proteasome-activating activity’, ‘peptidase activity’, ‘isoprenoid binding’, ‘hormone binding’, ‘endopeptidase activity’, ‘catalase activity’ and ‘abscisic acid binding’ (**Figure 4**). In GC-3, unique GO terms for molecular function were ‘enriched channel activity’ and ‘ammonia-lyase activity’. The GO analysis for down-regulated DEGs, resulted in 67, 37, and 22 enriched GO terms for molecular function in GC-2, GC-4, and GC-5, respectively (**Supplementary File 3**). No significant enrichment in GO terms for molecular function was observed in GC-3. Many of the GO terms for molecular function enriched for down-regulated in the susceptible cultivar (GC-2) were found to be up-regulated DEGs in resistant cultivars. The results of GO for molecular function by DEGs in different cultivars are represented in **Figure 4**.

**Figure 5:**
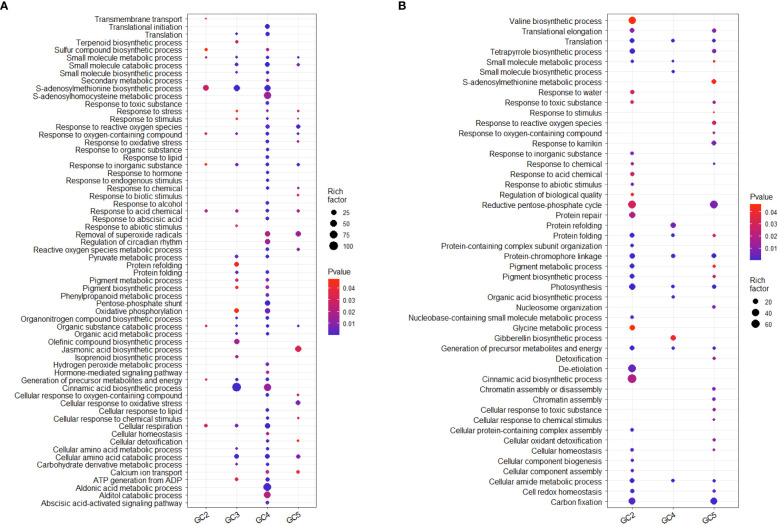
Overview of selected enriched GO terms for biological process **(A)** up-regulated and **(B)** down-regulated in transcriptome of cumin cultivars in response to *Foc* infection. The color shows p-value (Bejamini Hochberg) and size of the dots represents the rich factor of significant genes involved in each corresponding process. Rich factor is the ratio of the number of DEGs to the total number of genes in a given GO term. In GC-3 cultivar, no significant enrichment was observed for down-regulated DEGs hence not considered in the plot.

The results of GO analysis showed that total up-regulated significantly enriched terms for biological processes in GC-2, GC-3, GC-4, and GC-5 were 19, 91, 227, and 55, respectively and their details are given in **Supplementary File 4**. The enriched GO terms for biological processes in GC-4 were ‘secondary metabolic process’, ‘response to reactive oxygen species’, ‘response to organic substance’, ‘response to endogenous stimulus’, ‘phenylpropanoid metabolic process’, ‘hydrogen peroxide metabolic process’ and ‘hormone-mediated signaling pathway’. Compared to GC-4, GC-3 had less no. of uniquely up-regulated GO terms for biological processes i.e. ‘terpenoid synthetic process’, ‘response to abiotic stimulus’, ‘protein refolding’, ‘olefinic compound biosynthetic process’, and ‘isoprenoid biosynthetic process’. There were some commonly enriched GO terms for biological processes in GC-3 and GC-4, e.g. ‘translation’, ‘small molecule biosynthetic process’, ‘protein folding’, ‘oxidative phosphorylation’, ‘organic acid metabolic process’, and ‘cinnamic acid biosynthetic process’ (**Figure 5**). GO analysis for down-regulated DEGs, resulted in 118, 74, and 55 enriched GO terms for biological processes in GC-2, GC-4, and GC-5, respectively (**Supplementary File 4**). No significant enrichment in GO terms for biological processes was observed in GC-3. Some GO terms for biological processes like, ‘response to inorganic substance’, ‘response to chemical’, ‘response to abiotic stimulus’, ‘protein repair’, ‘pigment biosynthetic process’, ‘cinnamic acid biosynthetic process’ etc. were enriched for down-regulated DEGs in susceptible cultivar (GC-2) and most of these terms were up-regulated in resistant cultivars (**Figure 5**).

Considering all the up-regulated DEGs in different cultivars, 29 and 44 GO terms for cellular components were significantly enriched in resistant cultivars, GC-3 and GC-4, respectively. Considering up-regulated DEGs, no GO term for cellular components was significantly enriched in GC-2 and GC-5. In the case of down-regulated DEGs, 71, 4, 51, and 34 GO terms for cellular components were significantly enriched in GC-2, GC-3, GC-4, and GC-5, respectively (**Supplementary File 5**).

### 3.4 KEGG pathway enrichment analysis

**Figure 6:**
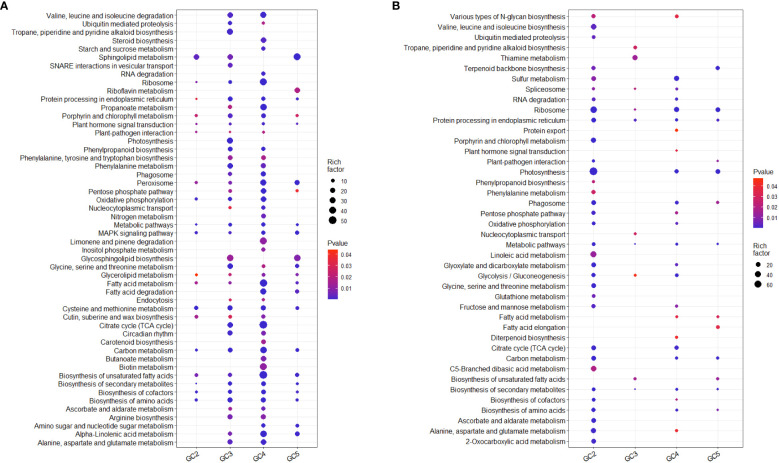
Enriched KEGG pathways of significant differentially expressed genes in cumin cultivars in response to Foc infection. **(A)** Up-regulated KEGG pathways and **(B)** Down-regulated KEGG pathways. The color of the dots represents the range of P-value and size represents the rich factor of significant genes involved in a pathway. Rich factor is the ratio of the number of DEGs to the total number of genes in a given KEGG pathway.

KEGG pathway analysis based on all the DEGs showed that 76 and 51 pathways were significantly up- and down-regulated, respectively in one or more cultivar(s) (**Supplementary Files 6, 7**). Many of them were probably directly or indirectly involved in plant defense against biotic or abiotic stresses in at least one of the cultivars. Considering the up-regulated DEGs, 28, 57, 65, and 30 KEGG pathways were enriched in GC-2, GC-3, GC-4, and GC-5, respectively. Among those enriched KEGG pathways, some metabolic pathways i.e. steroid biosynthesis, limonene and pinene degradation, butanoate metabolism, RNA degradation, starch and sucrose metabolism, aminoacyl-tRNA biosynthesis, fatty acid biosynthesis, inositol phosphate metabolism, biotin metabolism, and carotenoid biosynthesis were only enriched in GC-4 (resistant) and photosynthesis, tropane, piperidine, and pyridine alkaloid biosynthesis, SNARE interactions in vesicular transport, tyrosine metabolism, folate biosynthesis were only enriched in GC-3 (resistant) (**Figure 6**). Some pathways like phenylpropanoid pathway, phenylalanine metabolism, ubiquitin-mediated proteolysis, circadian rhythm, alanine, aspartate and glutamate metabolism, arginine biosynthesis, proteasome, phenylalanine, tyrosine and tryptophan biosynthesis, ascorbate and aldarate metabolism, propanoate metabolism, endocytosis, nucleocytoplasmic transport were enriched in GC-3 and GC-4 (**Figure 6**). The detailed results of KEGG pathway analysis for up-regulated DEGs of all the cultivars is shown in **Supplementary File 6**. The number of genes enriched in most of the individual pathways mentioned above was highest in GC-4, followed by GC-3, GC-5, and GC-2 (**Supplementary File 6**).

While considering down-regulated DEGs, 39, 11, 32, and 15 KEGG pathways were enriched in GC-2, GC-3, GC-4, and GC-5, respectively. Pathways enriched for down-regulated DEGs in GC-2 (susceptible) only were mostly enriched for up-regulated DEGs in resistant cultivars. The number of genes in enriched pathways was higher in susceptible cultivar (GC-2) compared to resistant or moderately resistant cultivars (**Supplementary File 7**).

### 3.5 Important DEGs involved in pathways related to disease-resistance

DEGs in different cultivars were used to identify some important genes that might be responsible for the response to the cumin wilt pathogen. Some of those genes from various important pathways have been given in **Table 1**.

**Table 1:** Important DEGs encoding transporters, receptors, enzymes and other proteins involved in immune response of cumin cultivars during *Foc* infection.

Gene id	Gene product	GC-2		GC-3		GC-4		GC-5	
		log2FC	p-value	log2FC	p-value	log2FC	p-value	log2FC	p-value
108227821	ABC transporter F family member 4	NA	NA	4.577859	0.034711	4.934982	0.013735	NA	NA
108218899	calcium-transporting ATPase 1, chloroplastic-like	NA	NA	NA	NA	5.645475	0.001975	NA	NA
108223191	calcium-transporting ATPase 4, endoplasmic reticulum-type	-4.40851	0.043068	NA	NA	5.797478	0.001232	NA	NA
108215612	probable aquaporin PIP2-4	3.18631	1.19E-09	5.314824	0.005642	6.645473	7.44E-05	1.628598	0.027587
108196057	respiratory burst oxidase homolog protein C-like	-4.40851	0.043068	2.194096	0.039435	1.676747	0.028498	5.77171	0.001924
108211593	protein DETOXIFICATION 27-like	NA	NA	NA	NA	5.282905	0.005582	NA	NA
108213173	ethylene-responsive transcription factor RAP2-1-like	NA	NA	NA	NA	5.999111	0.00065	NA	NA
108196505	linoleate 13S-lipoxygenase 3-1, chloroplastic-like	-9.73763	1.84E-10	7.122176	1.67E-05	4.934982	0.013735	-0.66888	1.48E-06
108223317	phenylalanine ammonia-lyase 1	-1.00648	0.022533	4.992896	0.013111	6.519942	0.000115	NA	NA
108195595	S-adenosylmethionine synthase 2	NA	NA	NA	NA	5.060513	0.010031	NA	NA
108206882	1-aminocyclopropane-1-carboxylate synthase	NA	NA	NA	NA	3.354853	0.009525	NA	NA
108209755	abscisic acid receptor PYL9	NA	NA	NA	NA	5.382441	0.004254	7.319192	7.89E-06
108211159	probable indole-3-acetic acid-amido synthetase GH3.1	NA	NA	NA	NA	5.282905	0.005582	NA	NA
108210264	auxin-responsive protein IAA27-like	NA	NA	NA	NA	2.011526	2.98E-08	NA	NA
108193147	glycerol kinase	-4.40851	0.043068	NA	NA	4.797478	0.018886	NA	NA
108209455	jasmonic acid-amido synthetase JAR1-like	NA	NA	NA	NA	-3.55222	0.000625	NA	NA
108211077	protein TIFY 10A-like	NA	NA	4.577859	0.034711	4.934982	0.013735	NA	NA
108211229	calcium-dependent protein kinase 20-like	NA	NA	NA	NA	7.833098	8.71E-07	NA	NA
108223506	calmodulin-binding receptor-like cytoplasmic kinase 3	NA	NA	NA	NA	5.999111	0.00065	NA	NA
108219484	probable calcium-binding protein CML49	NA	NA	NA	NA	5.382441	0.004254	NA	NA
108205673	coiled-coil domain-containing protein 130-like	NA	NA	NA	NA	2.733353	0.043683	NA	NA
108220227	nuclear transcription factor Y subunit B-1-like isoform X1	NA	NA	NA	NA	5.282905	0.005582	NA	NA
108202474	nucleoside diphosphate kinase 1	-1.9818	3.34E-05	-1.9818	3.34E-05	8.133757	2.59E-07	-1.17887	0.03751
108197695	probable LRR receptor-like serine/threonine-protein kinase PAM74	NA	NA	5.516457	0.003205	6.230436	0.000305	NA	NA
108194308	probable WRKY transcription factor 75	NA	NA	NA	NA	5.060513	0.010031	NA	NA
108201830	receptor-like protein kinase HSL1	-5.17405	0.006807	NA	NA	4.797478	0.018886	NA	NA
108205942	transcription factor MYB1R1-like	NA	NA	NA	NA	5.17599	0.007482	NA	NA
108196536	transcription factor MYC4	-5.4789	0.002822	NA	NA	2.120365	0.038344	NA	NA
108192395	transmembrane and coiled-coil domain-containing protein 4-like isoform X1	NA	NA	NA	NA	4.797478	0.018886	NA	NA
108220531	catalase isozyme 2-like	NA	NA	NA	NA	6.999109	2.1E-05	NA	NA
108192997	catalase-like	NA	NA	NA	NA	9.617751	4.58E-08	-6.51466	0.000297
108205636	L-ascorbate peroxidase, cytosolic-like	-7.42643	3.53E-06	1.15113	0.007786	5.47555	0.003249	1.137594	0.021058
108225244	peroxidase P7-like	NA	NA	3.679538	3.85E-05	6.723475	5.68E-05	NA	NA
108212562	superoxide dismutase [Cu-Zn] 2-like	NA	NA	-1.24064	0.000338	5.563013	0.002527	NA	NA
108219531	superoxide dismutase [Cu-Zn], chloroplastic	-2.20465	0.000474	NA	NA	3.162207	0.00073	1.69899	0.020069
108223791	superoxide dismutase [Mn], mitochondrial-like	1.100906	5.54E-05	1.073786	0.00066	5.645475	0.001975	-2.69349	0.013135
108208905	probable phospholipid hydroperoxide glutathione peroxidase	NA	NA	NA	NA	1.189313	0.020429	2.503096	2.38E-05
108197400	glutathione S-transferase DHAR2	NA	NA	NA	NA	4.934982	0.013735	NA	NA
108225144	alcohol dehydrogenase-like	-6.13098	0.000356	NA	NA	5.563013	0.002527	NA	NA
108192311	aldehyde dehydrogenase family 2 member B4, mitochondrial-like	NA	NA	NA	NA	5.060513	0.010031	NA	NA
108193625	pathogenesis-related protein A	-5.54602	0.002303	6.419159	0.000193	2.280834	8.60E-06	3.902452	0.000192
108226456	pathogenesis-related protein PR-1 type-like	7.258322	5.36E-05	9.543638	7.01E-10	8.841868	1.27E-08	8.579057	4.43E-08
108217674	pathogenesis-related protein PR-4-like	4.056700	1.06E-16	6.825783	4.78E-05	6.604831	8.59E-05	NA	NA
108217951	basic endochitinase-like	NA	NA	NA	NA	1.411396	0.047529	6.319196	0.000311
108193638	lignin-forming anionic peroxidase-like	NA	NA	NA	NA	4.558739	0.046786	NA	NA
108192978	polyphenol oxidase I, chloroplastic-like	-2.50192	0.010924	3.457141	0.009875	5.934981	0.000798	NA	NA
108218635	probable cinnamyl alcohol dehydrogenase 6 isoform X2	NA	NA	NA	NA	4.202862	0.000612	NA	NA
108208873	quinone oxidoreductase PIG3	NA	NA	2.035666	0.002217	5.282905	0.005582	NA	NA
108219257	tropinone reductase homolog	NA	NA	NA	NA	5.934981	0.000798	NA	NA
108209108	ubiquitin-activating enzyme E1 1-like	-4.40851	0.043068	2.405604	7.04E-15	1.535388	0.016436	-1.37152	0.015854
108215174	4-coumarate–CoA ligase-like 5	NA	NA	NA	NA	6.060512	0.000532	NA	NA
108200622	chalcone synthase 1	NA	NA	-1.34356	3.08E-14	2.617876	0.007482	5.3932	0.006198
108205090	chaperonin CPN60-2, mitochondrial	NA	NA	2.408223	0.020313	6.429745	0.000158	NA	NA
108219920	cytochrome P450 86A1 isoform X2	NA	NA	NA	NA	6.175989	0.000365	NA	NA
108202000	defensin-like protein 1	2.053927	1.12E-22	6.63675	9.19E-05	9.850586	1.23E-10	5.054939	2.04E-05
108202728	ferredoxin–nitrite reductase, chloroplastic isoform X1	-2.48525	0.001805	5.893494	0.008302	4.07733	0.000978	5.878625	0.001362
108196820	heat shock 70 kDa protein, mitochondrial-like	NA	NA	NA	NA	5.934981	0.000798	NA	NA
108210519	heat shock protein 83-like	NA	NA	NA	NA	6.282903	0.000258	NA	NA
108222698	heat shock protein 90-3 isoform X2	-4.67155	0.024420	4.899787	0.016568	5.934981	0.000798	-0.66469	0.009384
108209941	patatin-like protein 6	NA	NA	6.385211	0.000216	5.797478	0.001232	NA	NA
108202980	protein ECERIFERUM 1-like	NA	NA	5.693335	0.001909	4.354867	0.000342	NA	NA
108215375	probable mannitol dehydrogenase	NA	NA	NA	NA	8.040228	3.23E-06	-3.693518	0.006783
108214592	histone-lysine N-methyltransferase ATX4-like	NA	NA	4.452328	0.044907	7.200452	2.08E-05	NA	NA
108205079	histone-lysine N-methyltransferase setd3	-4.99348	0.011080	NA	NA	NA	NA	NA	NA
108222828	chromatin modification-related protein EAF1 B-like	NA	NA	NA	NA	5.563012	0.002527	NA	NA
108202994	ISWI chromatin-remodeling complex ATPase CHR11-like	NA	NA	NA	NA	2.733352	0.043682	NA	NA

DEGs, Differential expressed genes; *Foc*, *Fusarium oxysporum* f. sp. *cumini*; The healthy and infected samples of each cultivar were used to identify DEGs. The total DEGs were identified with a threshold of p-value ≤ 0.05 and with log2FC ≥ 2.0 and ≤ −2.0 for up- and down-regulated DEGs, respectively. NA, Not Applicable.

During the infection of *Foc* in cumin cultivars, some signal receptors and transporters for the transport of signals or molecules might have been involved in an immune response. Surface signal receptors or cytoplasmic receptors like ‘calcium-dependent protein kinase 20-like’ (108211229), ‘calmodulin-binding receptor-like cytoplasmic kinase’ (3108223506), ‘coiled-coil domain-containing protein 130-like’ (108205673), ‘nucleoside diphosphate kinase 1’ (108202474), ‘probable LRR receptor-like serine/threonine-protein kinase PAM74’ (108197695), ‘transmembrane and coiled-coil domain-containing protein 4-like isoform X1’ (108192395), and ‘receptor-like protein kinase HSL1’ (108201830) were up-regulated in GC-4. The DEGs for transporters were ‘ABC transporter F family member 4’ (108227821), ‘calcium-transporting ATPase 1, chloroplastic-like’ (108218899), ‘calcium-transporting ATPase 4, endoplasmic reticulum-type’ (108223191), ‘respiratory burst oxidase homolog protein C-like’ (108196057), and ‘protein DETOXIFICATION 27-like’ (108211593) which showed up-regulation in one or both of the resistant cultivars (GC-3 and GC-4). ‘Probable aquaporin PIP2-4’ (108215612) was up-regulated in all the cultivars but Log2FC was higher in GC-3 and GC-4 compared to the susceptible cultivar (**Table 1**). The activation of such signaling pathways altered gene expression which mainly depended on different transcription factors. Such differentially expressed transcription factors included ‘probable WRKY transcription factor 75’ (108194308), ‘transcription factor MYB1R1-like’ (108205942), ‘transcription factor MYC4’ (108196536), and ‘nuclear transcription factor Y subunit B-1-like isoform X1’ (108220227) which were up-regulated in GC-4 only.

As a result of the immune response towards pathogen infection, ROS and H_2_O_2_ flux increased which was scavenged by different antioxidant enzymes in the cell. The DEGs for antioxidant enzymes i.e. ‘catalase isozyme 2-like’ (108220531), ‘catalase-like’ (108192997), ‘superoxide dismutase [Cu-Zn] 2-like’ (108212562), ‘superoxide dismutase [Cu-Zn], chloroplastic’ (108219531), ‘glutathione S-transferase DHAR2’ (108197400), and ‘probable phospholipid hydroperoxide glutathione peroxidase’ (108208905) showed up-regulation in GC-4 only. Expression of the other two ROS scavenging enzymes ‘L-ascorbate peroxidase, cytosolic-like’ (108205636) and ‘peroxidase P7-like’ (108225244) showed up-regulation in GC-3 and GC-4. ‘Superoxide dismutase [Mn], mitochondrial-like’ (108223791) showed up-regulation in all the cultivars.

The expression profiles of different genes involved in pathways of stress-related hormones like jasmonic acid (JA), salicylic acid (SA), ethylene, and abscisic acid (ABA) showed up-regulation of ‘ethylene-responsive transcription factor RAP2-1-like’ (108213173), ‘linoleate 13S-lipoxygenase 3-1, chloroplastic-like’ (108196505), ‘phenylalanine ammonia-lyase 1’ (108223317), ‘S-adenosylmethionine synthase 2’ (108195595), ‘1-aminocyclopropane-1-carboxylate synthase’ (108206882), ‘abscisic acid receptor PYL9’ (108209755), ‘glycerol kinase’ (108193147), ‘jasmonic acid-amido synthetase JAR1-like’ (108209455), and ‘protein TIFY 10A-like’ (108211077) in one or both of the resistant cultivars (**Table 1**).

Some DEGs involved in defense response including ‘glutathione S-transferase DHAR2’ (108197400), ‘alcohol dehydrogenase-like’ (108225144), ‘endochitinase-like’ (108217951), ‘lignin-forming anionic peroxidase-like’ (108193638), ‘polyphenol oxidase I, chloroplastic-like’ (108192978), ‘probable cinnamyl alcohol dehydrogenase 6 isoform X2’ (108218635), ‘quinone oxidoreductase PIG3’ (108208873), ‘tropinone reductase homolog’ (108219257), ‘ubiquitin-activating enzyme E1 1-like’ (108209108), ‘4-coumarate–CoA ligase-like 5’ (108215174), ‘chalcone synthase 1’ (108200622), ‘chaperonin CPN60-2, mitochondrial’ (108205090), ‘cytochrome P450 86A1 isoform X2’ (108219920), ‘ferredoxin–nitrite reductase, chloroplastic isoform X1’ (108202728), ‘heat shock 70 kDa protein, mitochondrial-like’ (108196820), ‘heat shock protein 83-like’ (108210519), ‘patatin-like protein 6’ (108209941), ‘protein ECERIFERUM 1-like’ (108202980), probable mannitol dehydrogenase (108215375) etc. were up-regulated in GC-4 and might have involved in different pathways for secondary metabolite synthesis, cell wall strengthening and pathways for enhancing pathogen resistance in plant. Among those DEGs, defensin-like protein 1 (108202000) was up-regulated in all the cultivars but the Log2FC was higher in resistant cultivars compared to the susceptible cultivar (**Table 1**).

The change in the expression of genes providing defense against pathogens might be due to epigenetic changes occurring in GC-4. Some enzymes involved in histone modification e.g. ‘histone-lysine N-methyltransferase ATX4-like’ (108214592) (catalyzes H3K4me3) was up-regulated in GC-4 whereas ‘histone-lysine N-methyltransferase setd3’ (108205079) (catalyzes H3K4me3) was down-regulated in susceptible cultivar (GC-2). Moreover, enzymes involved in chromatin modification e.g. ‘chromatin modification-related protein EAF1 B-like’ (108222828) (involvement in histone acetyltransferase complex) and ‘ISWI chromatin-remodeling complex ATPase CHR11-like’ (108202994) (possesses intrinsic ATP-dependent nucleosome-remodeling activity) were up-regulated in GC-4 only. The methylation, acetylation, and chromatin remodeling pattern might have altered the expression of genes involved in defense against the pathogen.

## 4 Discussion

Fusarium wilt caused by many forms of soil-borne pathogen *F. oxysporum* is a widespread plant disease. Several hundred plant species are susceptible to *F. oxysporum*, including economically important food crops like cumin, coriander, legumes, vegetables, and melons. In the recent years, transcriptome analysis has been performed in different crops like sesame (Wei et al., 2016), banana (Sun et al., 2019; Dong et al., 2020), flax (Boba et al., 2021), cotton (Hou et al., 2021), some pulse crops like mung bean (Chang et al., 2021) and common bean (Leitão et al., 2021) to elucidate the enrichment of pathways or genes for fusarium wilt resistance. Since the cumin defense mechanism against wilt pathogens has not yet been revealed, we attempted to recognize the genes and pathways associated with fusarium wilt resistance in different cultivars by comparing their transcriptome.

Mechanisms of resistance to *F. oxysporum* are very complex and a network of phytohormone signaling (Boba et al., 2021). In the present study, some defense-related genes and pathways have been enriched during *Foc* infection. Among the enriched GO terms for biological processes in GC-4, some of them have been reported to be enriched in plants during *Fusarium* infection such as ‘secondary metabolic process’ (Kaushal et al., 2021), ‘response to reactive oxygen species’ (Chang et al., 2021), ‘response to organic substance’ (Toueni et al., 2016), ‘response to endogenous stimulus’ (Xiong et al., 2021), ‘phenylpropanoid metabolic process’ (Zhu et al., 2021), ‘hydrogen peroxide metabolic process’ (Zhang et al., 2019) and ‘hormone-mediated signaling pathway’ (Zhang et al., 2018) considering up-regulated DEGs.

Considering up-regulated DEGs, more KEGG pathways were enriched in GC-4 followed by GC-3, GC-5, and GC-2. The steroid biosynthesis pathway was enriched in GC-4. In this pathway, genes up-regulated in one or both of the resistant cultivars in the present study, have an important role in biotic stress resistance i.e. fungal infection in plants (Kong et al., 2005; Lu et al., 2020; Pandian et al., 2020). Butanoate metabolism pathway enriched in only GC-4 possessed genes that were reported to be up-regulated during *F. oxysporum* f. sp. *vasinfectum* infection in cotton (Yao et al., 2019). ‘Glutamate decarboxylase’ increases gamma-aminobutyric acid (GABA) accumulation which has been observed during various plant-pathogen interactions and has been associated with disease resistance response against tomato pathogen *Ralstonia solanacearum* (Wang et al., 2019a). ‘Probable enoyl-CoA hydratase 1, peroxisomal’ is involved in β-oxidation of fatty acids which produces cytotoxic ROS as byproducts (Yu et al., 2019). The fatty acid biosynthesis pathway was significantly enriched in resistant cultivars which is in agreement with its enrichment observed in pepper during fusarium wilt (Zhu et al., 2021). Genes up-regulated in resistant cultivars were involved in enhancing resistance against fungal diseases in plants (Lee et al., 2021; Peng et al., 2022). Up-regulated DEGs in the inositol phosphate metabolism pathway in GC-4 were also found to be up-regulated in the previous studies e.g. in pepper during *Fusarium* infection (Zhu et al., 2021).

In GC-4, some genes involved in disease resistance were up-regulated which might have provided resistance against *Foc*. Up-regulation of these genes during fungal infection have been supported by some previous reports like, LRR-RLK in resistant rubber tree against *Corynespora cassiicola* (Roy et al., 2019), transcription factors WRKY, and MYB in apple plant showing resistance against *Fusarium proliferatum* f. sp. *malus domestica* (Duan et al., 2022), superoxide dismutase, catalase, ascorbate peroxidase and monodehydroascorbate reductase in resistant plant of cucumber against the infection of *Alternaria cucumerina* (Sa et al., 2022), ascorbate and carotenoids in pepper against *Fusarium* infection (Zhu et al., 2021), calcium-dependent protein kinase (CDPK) and R-proteins in banana resistant plant against wilt pathogen (Sun et al., 2019), calmodulin (CaM) and calmodulin-like protein (CML) in wild cabbage resistant to *Plasmodiophora brassicae* during infection (Zhang et al., 2016), pathogenesis related protein-1 (PR-1) and PR-2 in rose in response to powdery mildew (Chandran et al., 2021), in tomato plants infected with *F. oxysporum* (Slezina et al., 2021), chitinase and glucanase in apple plant resistant to *Fusarium* infection (Duan et al., 2022), defensin-like protein 1 in tomato plants infected with *F. oxysporum* (Slezina et al., 2021) and lignin biosynthesis in cotton during *Fusarium* wilt infection (Hou et al., 2021). Several ROS scavenging enzymes were more expressed in GC-4 during *Foc* infection. Our findings were in agreement with the findings of the previous study related to Alternaria leaf spot resistance in cucumber (Sa et al., 2022). Catalase as a major H_2_O_2_-scavenging enzyme is also widely involved in plant immunity (Yan et al., 2021). Over-accumulation of ROS, a result of pathogen infection, probably leads to chlorotic and membrane lipid peroxidation (Sa et al., 2022).

During *Foc* infection in GC-4, interactions among different defense hormones might have a crucial role in resistance against the pathogen. ‘Probable indole-3-acetic acid-amido synthetase GH3.1’ is an auxin-responsive gene that acts in the auxin-dependent development of plants for activating biotic stress resistance pathways independent of salicylic acid signaling and jasmonic acid signaling which enhances resistance to both fungal and bacterial pathogens (Ding et al., 2008). ‘Auxin-responsive protein IAA27-like’ regulates the auxin and ethylene signaling pathways and it is also involved in the regulation of strigolactone biosynthesis. Strigolactones are plant hormones and root-derived signals that regulate shoot branching and respond against parasitic and symbiotic interactions ultimately enhancing plant growth and diminishing the effects of different stresses (Iqbal and Peng, 2022). ‘1-aminocyclopropane-1-carboxylate (ACC) synthase’ which is involved in ethylene biosynthesis by catalyzing the conversion of S-adenosyl-L-methionine (SAM) into ACC was up-regulated in GC-4 only. In a past study, its involvement in ethylene biosynthesis and signaling for disease resistance probably by activating the production of ROS and phytoalexins in rice during *M. oryzae* infection was reported (Yang et al., 2017). Overexpression of ‘abscisic acid receptor PYL9’ induced the elongation of lateral roots in the presence of abscisic acid (ABA) and recovery of lateral roots from ABA inhibition via MYB transcription factors (Xing et al., 2016). In the present study, ‘abscisic acid receptor PYL9’ was significantly up-regulated in GC-4 only that indicated the possible mechanism of resistance in GC-4. ‘Jasmonic acid-amido synthetase JAR1-like’ was down-regulated and ‘linoleate 13S-lipoxygenase 3-1, chloroplastic-like’ were up-regulated in GC-4 in the present study and involved in jasmonic acid pathway. These genes might have been associated with the downstream JA-responsive resistance genes (Luo et al., 2019). ‘Phenylalanine ammonia-lyase 1’ gene is involved in salicylic acid pathway and was up-regulated in GC-4. SA synthesis and action have been induced according to pathogen-associated molecular patterns (PAMPs) and play an important role in immune responses in plants against fungal pathogens (Luo et al., 2019).

In the present study, we also found the up-regulation of several defense-related genes. ‘Pathogenesis-related protein PR-1 type-like’ was significantly up-regulated in all the cultivars which are among the most abundantly produced proteins in plants during pathogen infection. Its expression has been considered a sign of salicylic acid-mediated disease resistance. PR-1 has broad antimicrobial activity and its overexpression in plants results in increased resistance to fungi and bacteria (Breen et al., 2017). ‘Basic endochitinase-like’ protein was up-regulated in GC-4. Interaction between plant and pathogen directs the rapidity of chitinase (a type of PR-proteins) induction in plant tissues (Vaghela et al., 2022). The expression of ‘lignin-forming anionic peroxidase-like’ was increased in GC-4. It is an important enzyme in lignin biosynthesis which results in secondary cell wall synthesis ultimately providing a physical barrier for pathogens to enhance plant resistance. It was in agreement with the results found in the past in cotton during *Fusarium* infection (Yao et al., 2019). The probable mannitol dehydrogenase gene was up-regulated in GC-4 only which might be involved in pathogen-secreted mannitol catabolism (Meena et al., 2015). Some genes i.e. ‘glycerol kinase’ and ‘heat shock protein 90-2-like’ were up-regulated in GC-4 and down-regulated in GC-2. These genes were reported to be up-regulated in resistant plants to provide resistance against pathogens (Li et al., 2021; Xiao et al., 2022). The gene for ‘respiratory burst oxidase homolog protein C-like’ (RBOHC) was up-regulated in GC-3, GC-4, and GC-5 and down-regulated in GC-2. Similarly, it was reported that in *Arabidopsis* it was up-regulated for resistance against *Botrytis cinerea* (van Rensburg et al., 2020).

Cutin, suberine, and wax biosynthesis pathway was significantly enriched in all the cultivars except GC-5. In the recent past, it was observed that it was significantly involved in resistance against *Fusarium verticillioides* infection in sugarcane by the KEGG pathway enrichment analysis (Wang et al., 2019b). ‘Protein ECERIFERUM 1-like’ promotes long-chain alkane wax biosynthesis to enhance plant response to biotic and abiotic stresses (Bourdenx et al., 2011). ‘Omega-hydroxypalmitate O-feruloyl transferase-like’ is possibly involved in cutin and suberin biosynthesis and the increase in its transcripts might be an indication of a strengthening of the barrier against the fungal pathogen (Balestrini et al., 2020; Shi et al., 2021). Hence, the up-regulation of some of the genes involved in immune response in resistant cultivars indicated that they might be crucial in providing resistance against wilt pathogens in cumin.

The variation in epigenetic changes in resistant and susceptible cultivars might have played a crucial role in the defense response against pathogens due to the variable expression of different genes. Enzymes involved in histone modification e.g. ‘histone-lysine N-methyltransferase ATX4-like’ (catalyzes histone H3 lysine 4 trimethylation (H3K4me3)) were up-regulated in GC-4 whereas ‘histone-lysine N-methyltransferase setd3’ (catalyzes H3K4me3) was down-regulated in susceptible cultivar (GC-2) only. The enrichment of H3K4me3 at transcription start sites (TSSs) promotes transcription by recruiting PHD-domain-containing proteins (e.g. TATA-box-binding protein associated factor 3 (TAF3)) involved in transcription initiation (Wang et al., 2023). H3K4me is catalyzed by a conserved protein complex (COMPASS-like complex) and is mainly located in euchromatin (Xie and Duan, 2023). Generally, H3K4me3 is associated with transcriptionally active regions hence, the methylation pattern might have activated the genes involved in defense against the wilt pathogen in GC-4. Enzymes involved in chromatin modification e.g. ‘chromatin modification-related protein EAF1 B-like’ (involvement in histone acetyltransferase complex) and ‘ISWI chromatin-remodeling complex ATPase CHR11-like’ (108202994) (possesses intrinsic ATP-dependent nucleosome-remodeling activity) were up-regulated in GC-4 only in the present study. ‘Chromatin modification-related protein EAF1 B-like’ is a component of the NuA4 histone acetyltransferase complex. This complex acetylates nucleosomal histones H2A and H4 for the transcriptional activation of related genes. The acetylation of histones controlled the expression of different developmental stages in plants (Bieluszewski et al., 2015). ‘ISWI chromatin-remodeling complex ATPase CHR11-like’ exhibits intrinsic ATP-dependent nucleosome-remodeling activity. Chromatin-remodeling factors regulate the transcription initiation at many developmental stages of the plant life cycle (Huanca-Mamani et al., 2005). The epigenetic changes like methylation, acetylation, and chromatin remodeling patterns in GC-4 might have altered the expression of genes involved in controlling developmental stages and stress responses for defense against the wilt pathogen.

**Figure 7:**
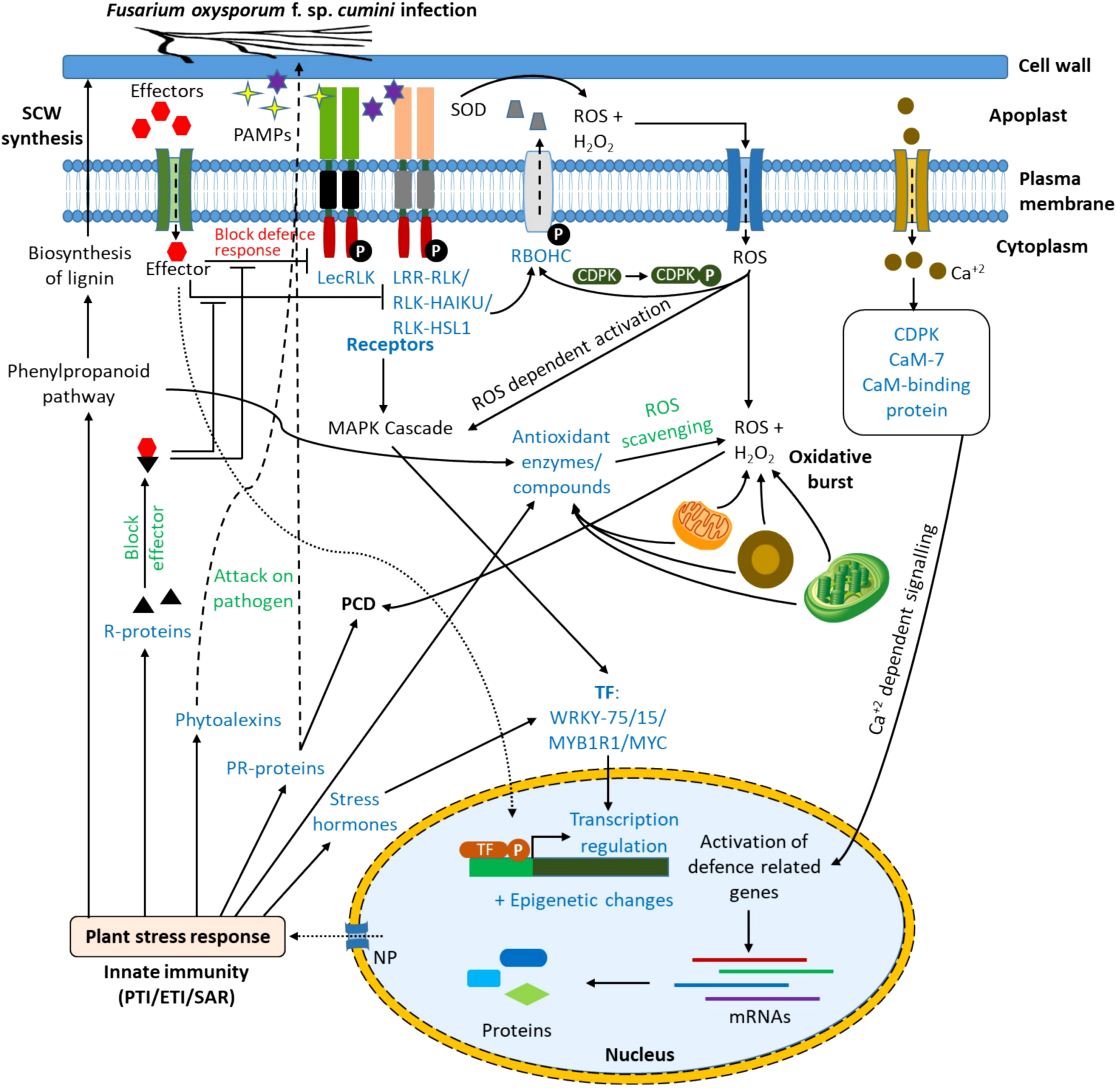
Response in resistant cultivars of cumin to *F. oxysporum* f. sp. *cumini* infection (CaM, Calmodulin; CDPK, Calcium dependent protein kinase; CML, Calmodulin-like protein; ETI, Effector triggered immunity; LecRLK, Lectin receptor-like kinases; LRR-RLK, Leucine-rich repeat receptor-like kinases; NP, Nuclear pore; PAMPs, Pathogen-associated molecular patterns; PCD, Programmed cell death; PR-protein, Pathogenesis-related protein; PTI, Pattern-triggered immunity; RBOHC, Respiratory burst oxidase homolog protein C (Calcium-dependent NADPH oxidase); RLK-HAIKU2, Receptor-like kinase- HAIKU2 domain; RLK-HLS1, Receptor-like kinase- HLS1 domain; ROS, Reactive oxygen species; R-protein, Resistance protein; SAR, Systemic acquired resistance; SCW, Secondary cell wall; SOD: Superoxide dismutase; TF, Transcription factor).

From the results of DEGs, KEGG, and GO enrichment analysis, the probable immune response to the wilt pathogen, *Foc* in the resistant cultivar (GC-4) has been proposed (**Figure 7**). The PAMPs might be detected by the plant receptors. These receptors possessing a kinase domain ultimately activated different signaling pathways like the mitogen-activated protein kinase (MAPK) signaling pathway in the cytoplasm (Nag et al., 2022). It activated some transcription factors (e.g. WRKY75 and MYB1R1) which in turn, up-regulated defense-related genes and pathways in the nucleus. The detection of PAMPs by receptors might have elicited ROS in the apoplastic region by the induction of NADPH oxidases, polyamine oxidases, and peroxidases. The elevated level of ROS and H_2_O_2_ might be scavenged by the up-regulation of ROS scavenging enzymes. The production of H_2_O_2_ and ROS from mitochondria, chloroplast, and peroxisomes in response to the pathogen attack initiated different signaling pathways toward the resistance against the pathogen. The ROS production in the apoplastic region might have a role in cell wall loosening and cross-linking. Those ROS might also enter into the cytoplasm via aquaporins (e.g. plasma membrane intrinsic protein (PIP2-4)) where it also induces ROS-dependent activation of the MAPK cascade. It also activated calcium channels on the plasma membrane which increased the Ca^+2^ concentration in the cytoplasm activating CDPKs and calmodulin-like protein. These CDPKs might have relayed the signals to the nucleus of the cell which leads to altering the gene expression of important defense-related genes and pathways. The effector molecules secreted by pathogens might have been recognized by the receptors from R-proteins and they might have blocked the effectors’ activity to block the induction of signaling pathways by kinases after pathogen-associated molecular pattern molecules (PAMPs) detection. Effectors might activate immune responses in plants via different signaling pathways (Jose et al., 2020; Nag et al., 2022). Pattern-triggered immunity (PTI) and effector-triggered immunity (ETI) activated defense signaling pathways inducing expression of PR-proteins (e.g. chitinase, peroxidases, etc.), phytoalexins, R-proteins, and some defense-related pathways as mentioned in results. The phenylpropanoid pathway is involved in the biosynthesis of lignin and ultimately secondary cell wall (SCW) synthesis, hence providing a physical barrier to pathogen entry in the plant cell (Yao et al., 2019). Phytoalexins and some PR-proteins attack the pathogen and reduce the infection. The activation of signaling pathways enhanced the expression of stress-responsive phytohormones (e.g. SA, JA, and ABA) which regulated the expression of other stress-responsive genes (Ali et al., 2018). Some of the PR-proteins might have been involved in programmed cell death (PCD) (Shao et al., 2021). The overall initiation for expression of various genes in response to the infection might be due to epigenetic changes like histone methylation, histone acetylation, and chromatin remodeling. The probable mechanism of defense in wilt GC-4 against *F. oxysporum* f. sp. *cumini* is shown in **Figure 7**. The activation or up-regulation of different genes and pathways related to defense possibly provided resistance in the GC-3 and/or GC-4 cultivars.

## 5 Conclusion

The transcriptome analysis of wilt-resistant and susceptible cultivars of cumin indicated the variation in the regulation of different pathways and genes by altering signaling pathways and immune system-related pathways. The up-regulation of receptors (e.g. RLKs) for recognition of PAMPs, transcription factors, ROS generating and ROS scavenging enzymes, Ca^+2^ transporters and receptors, R-proteins, PR-proteins and, phytoalexins in GC-4 might have provided resistance against *Foc*. Some important pathways enriched in GC-4 and GC-3 were the phenylpropanoid pathway, TCA cycle, phenylalanine metabolism, ubiquitin-mediated proteolysis, ascorbate and aldarate metabolism, and propanoate metabolism. Pathways like steroid biosynthesis, starch and sucrose metabolism, fatty acid biosynthesis, butanoate metabolism, inositol phosphate metabolism, limonene, and pinene degradation, and carotenoid biosynthesis were enriched for up-regulated DEGs in GC-4. The activation or up-regulation of different genes and pathways related to the defense provided resistance in the GC-4 cultivar. These results provide insights to develop cumin cultivars resistant to wilt, in addition to allowing the investigation of the detailed mechanisms underlying cumin defense responses against *Foc*.

## Data availability statement

The datasets presented in this study can be found in online repositories. The names of the repository/repositories and accession number(s) can be found in the article/**Supplementary Material**.

## Author contributions

DTD: wrote the first draft of the manuscript, literature review, and transcriptome data analysis; NS: drafted the bioinformatics pipeline for data analysis and transcriptome data analysis; MP: sample collection, library preparation, and sequencing; MJ: guidance in the data analysis and manuscript proof-reading; AKP: guidance in the data analysis and manuscript proof-reading; CGJ: conception and design of the study, guidance in the data analysis and manuscript proof-reading. All authors contributed to the article and approved the submitted version.
