# Supplementary figures and images for: Resistant cumin cultivar, GC-4 counters Fusarium oxysporum f. sp. cumini infection through up-regulation of steroid biosynthesis, limonene and pinene degradation and butanoate metabolism pathways
Source: Front Plant Sci. 2023 Oct 17;14:1204828. doi: 10.3389/fpls.2023.1204828 (PMC10616826; doi:10.3389/fpls.2023.1204828)

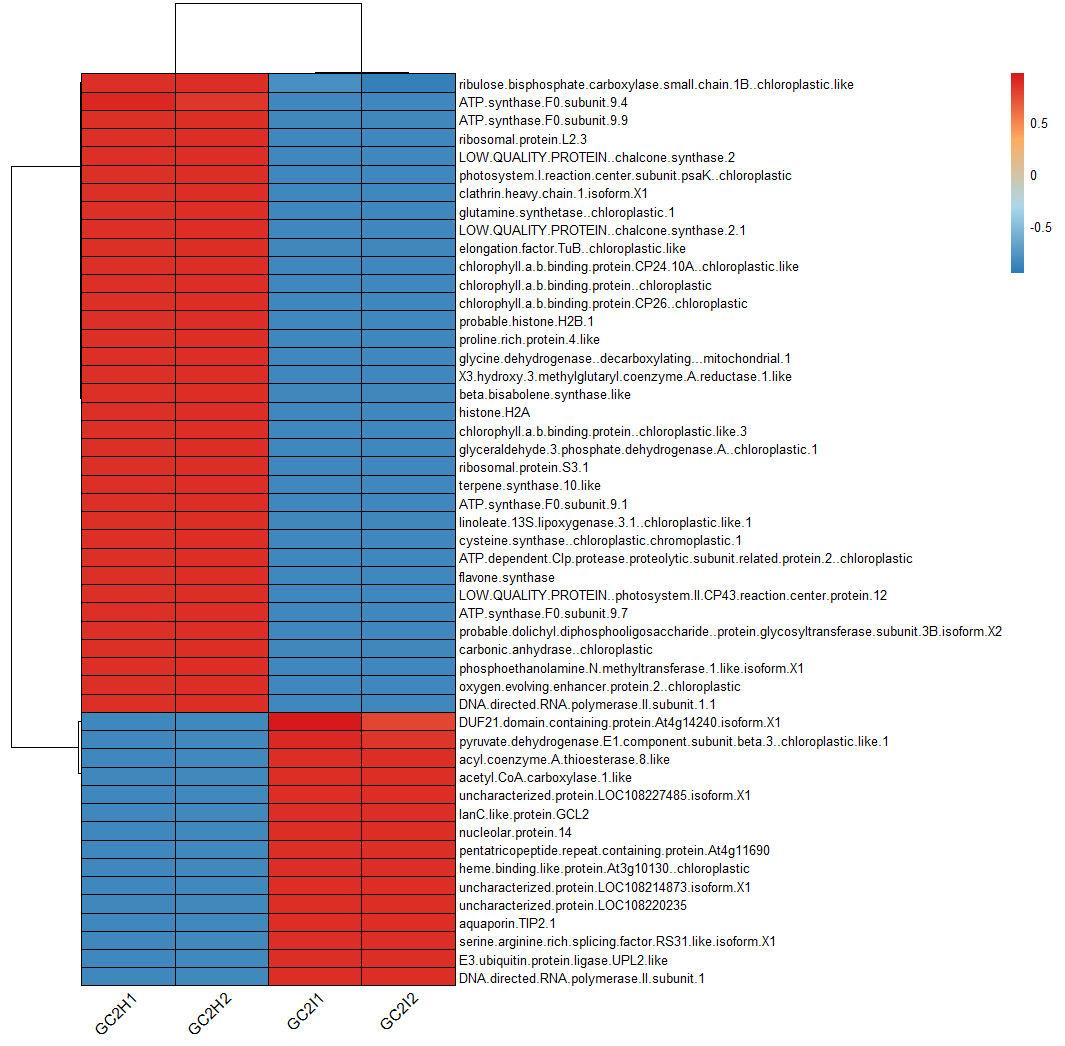

Supplement: Supplementary file 1 [file Image_1.tiff]

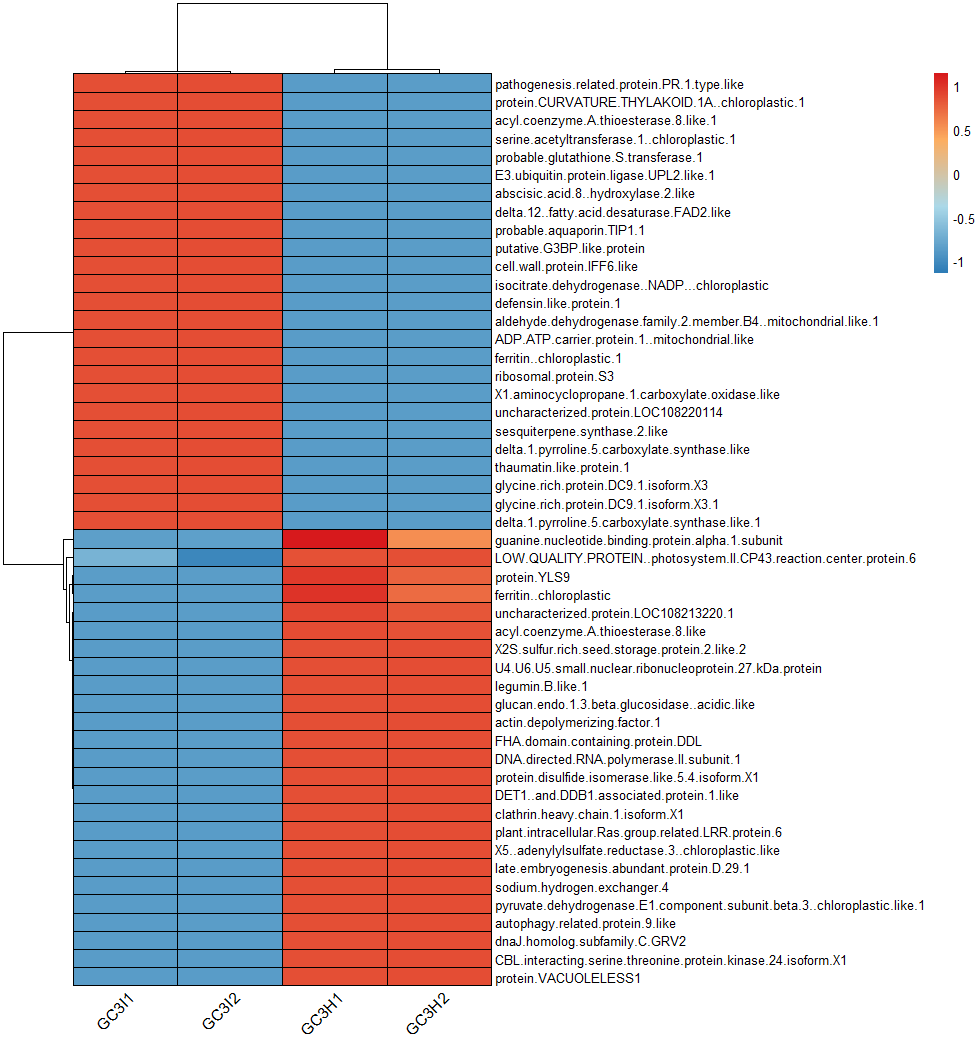

Supplement: Supplementary file 2 [file Image_2.tiff]

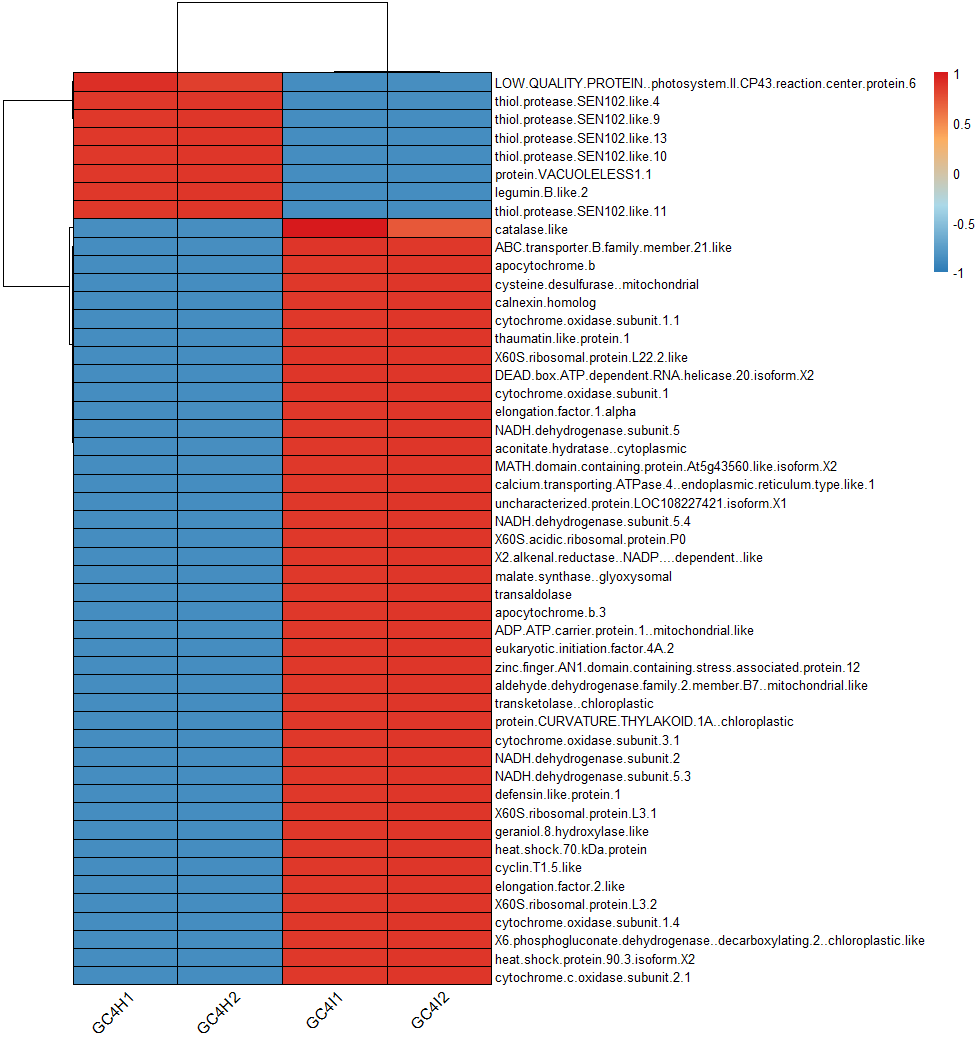

Supplement: Supplementary file 3 [file Image_3.tiff]

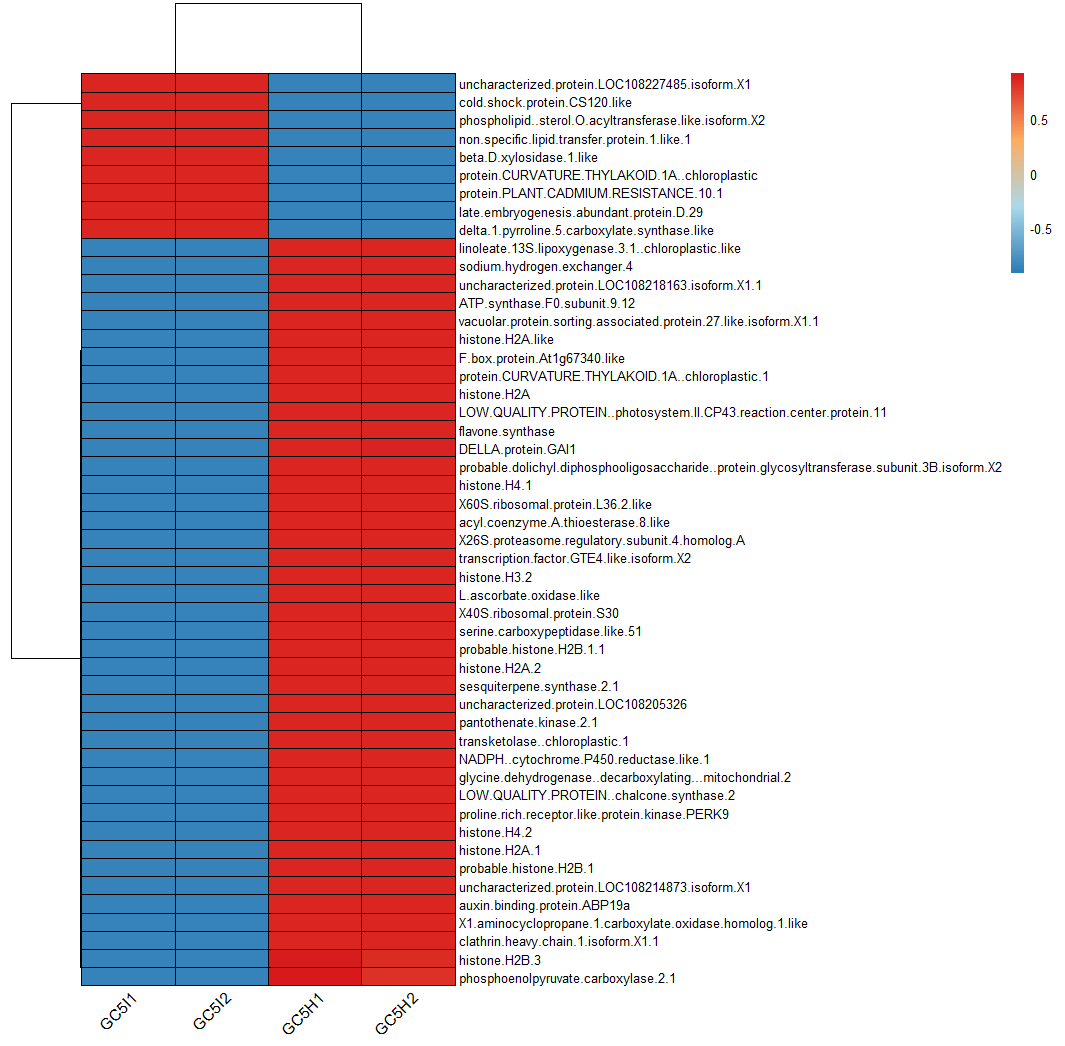

Supplement: Supplementary file 4 [file Image_4.tiff]
