# Supplementary material for: Resistant cumin cultivar, GC-4 counters Fusarium oxysporum f. sp. cumini infection through up-regulation of steroid biosynthesis, limonene and pinene degradation and butanoate metabolism pathways
Source: Front Plant Sci. 2023 Oct 17;14:1204828. doi: 10.3389/fpls.2023.1204828 (PMC10616826; doi:10.3389/fpls.2023.1204828)
Supplement: Supplementary file 5 [file Presentation_1.pdf]

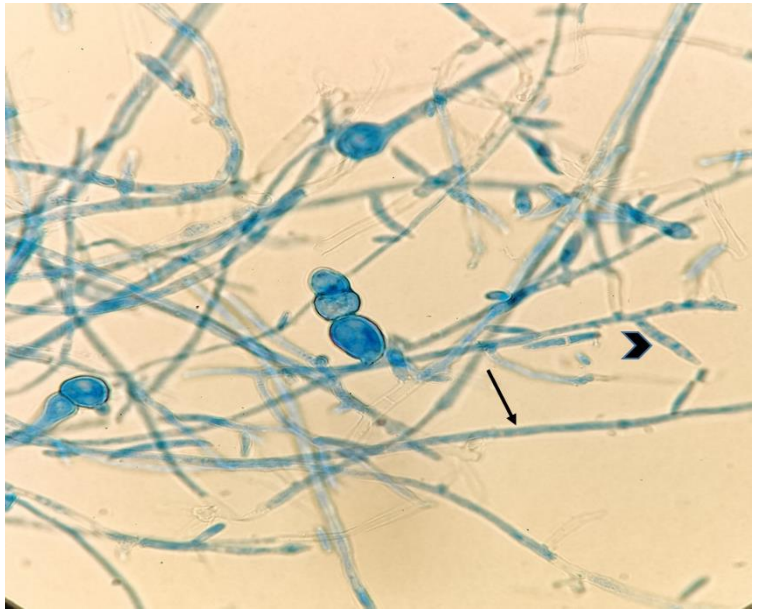

Microscopy image of *Fusarium oxysporum* showing mycelia and conidiospores (40x). Conidiospores (arrow head) and mycelium (arrow)

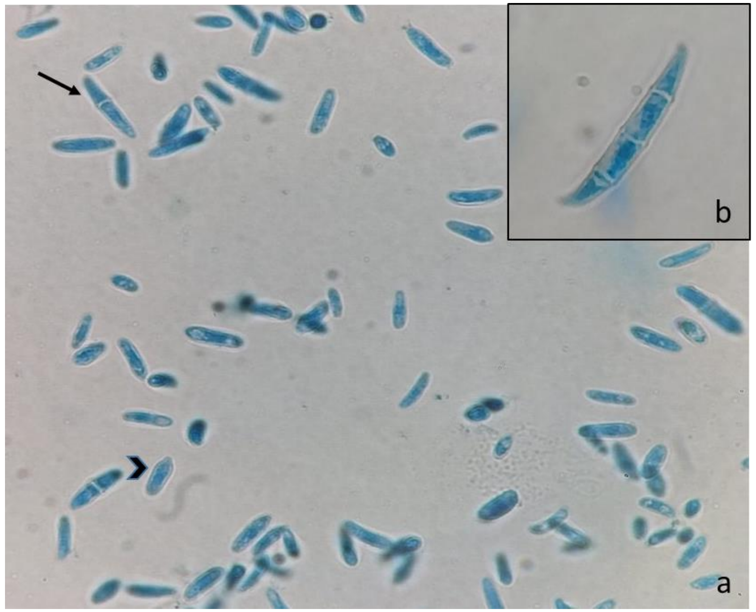

Microscopy images of *Fusarium oxysporum* showing conidiospores .
a) Macroconidiospores (arrow) and microconidiospores (arrow head) (40x) b) Macroconidiospore (enlarged)(100x)

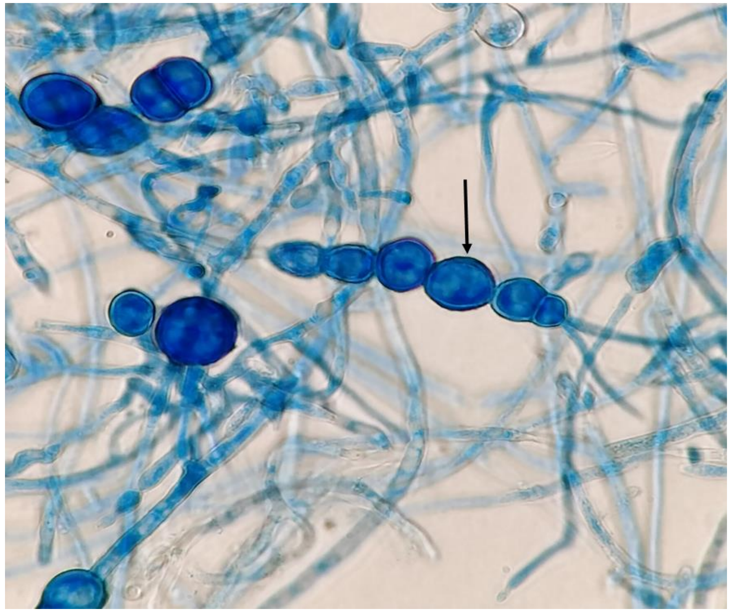

Microscopy image of *Fusarium oxysporum* showing mycelia and Chlamydospore (Arrow) (100x)

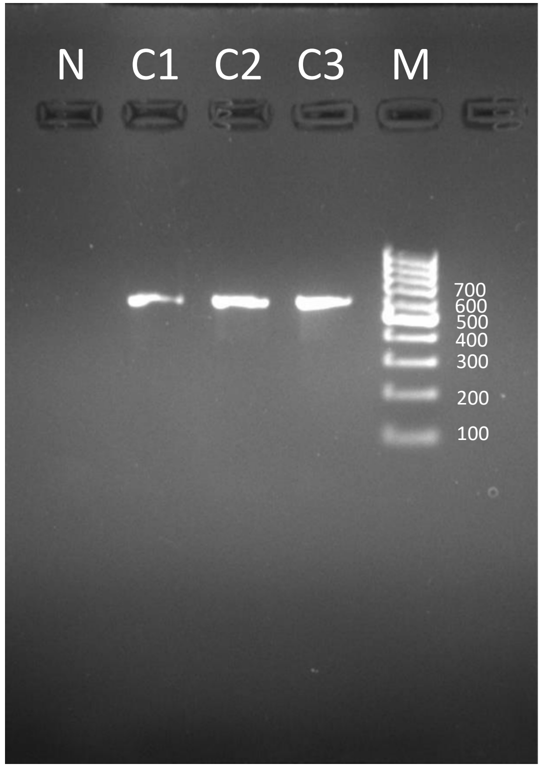

Results of ITS (ITS1 and ITS4 primers) amplification products separated on agarose gel (2%). N: Non template control; C1-C3: Fungal sample isolated from infected plant; M: 100bp DNA ladder

Sanger sequencing

>ITS1 primer
GGATCATTACCGAGTTTACAACTCCCAAACCCCTGTGAACATACCACTTGTTGCCTCGGCGGATCAGCCCGC
TCCCGGTAAAACGGGACGGCCCGCCAGAGGACCCCTAAACTCTGTTTCTATATGTAACTTCTGAGTAAAACC
ATAAATAAATCAAAACTTTCAACAACGGATCTCTTGGTTCTGGCATCGATGAAGAACGCAGCAAAATGCGATA
AGTAATGTGAATTGCAGAATTCAGTGAATCATCGAATCTTTGAACGCACATTGCGCCCGCCAGTATTCTGGCG
GGCATGCCTGTTCGAGCGTCATTTCAACCCTCAAGCACAGCTTGGTGTTGGGACTCGCGTTAATTCGCGTTC
CTCAAATTGATTGGCGGTCACGTCGAGCTTCCATAGCGTAGTAGTAAAACCCTCGTTACTGGTAATCGTCGC
GGCCACGCCGTTAAACCCCAACTTCTGAATGTTGACCTCGGATCAGGTAGGAATACCCGCTGAACTTAAGCA
T
>ITS4 Primer
GTCACATTCAGAAGTTGGGGTTTAACGGCGTGGCCGCGACGATTACCAGTAACGAGGGTTTTACTACTACGC
TATGGAAGCTCGACGTGACCGCCAATCAATTTGAGGAACGCGAATTAACGCGAGTCCCAACACCAAGCTGTG
CTTGAGGGTTGAAATGACGCTCGAACAGGCATGCCCGCCAGWATACTGGCGGGCGCAATGTGCGTTCAAAG
ATTCGATGATTCACTGAATTCTGCAATTCACATTACTTATCGCATTTTGCTGCGTTCTTCATCGATGCCAGAAC
CAAGAGATCCGTTGTTGAAAGTTTTGATTTATTTATGGTTTTACTCAGAAGTTACATATAGAAACAGAGTTTAG
GGGTCCTCTGGCGGGCCGTCCCGTTTTACCGGGAGCGGGCTGATCCGCCGAGGCAACAAGTGGTATGTTC
ACAGGGGTTTGGGAGTTGTAAACTCGGTAATGATCCCTCCGCTGGTTC

NCBI blastn results of fungal cultures isolated from infected plant

Identified species	Max Score	Total Score	Query Cover	E value	Per. Ident	Acc. Len	Accession
Fusarium oxysporum	937	937	100%	0.0	100.00%	568	MT530243.1
