# Supplementary material for: Resistant cumin cultivar, GC-4 counters Fusarium oxysporum f. sp. cumini infection through up-regulation of steroid biosynthesis, limonene and pinene degradation and butanoate metabolism pathways
Source: Front Plant Sci. 2023 Oct 17;14:1204828. doi: 10.3389/fpls.2023.1204828 (PMC10616826; doi:10.3389/fpls.2023.1204828)
Supplement: Supplementary file 12 [file Table_1.docx]

***Supplementary Material***

**Resistant cumin cultivar, GC-4 counters *Fusarium oxysporum* f. sp. *cumini* infection through up-regulation of steroid biosynthesis, limonene and pinene degradation and butanoate metabolism pathways**

**Authors:** Darshan T. Dharajiya, Nitin Shukla, Maharshi Pandya, Madhvi Joshi*, Amrutlal K. Patel*, Chaitanya G. Joshi*

**Affiliation:** Gujarat Biotechnology Research Centre (GBRC), Department of Science and Technology, Government of Gujarat, Gandhinagar- 382010, Gujarat, India.

***Correspondence**

Madhvi Joshi: jd1@gbrc.res.in

Amrutlal K. Patel: jd2@gbrc.res.in

Chaitanya G. Joshi: director@gbrc.res.in

**1. Supplementary data (.xlsx files)**

**Supplementary File 1:** Identification of fungal culture isolated from infected plant by microscopic observation and sequencing and analysis of ITS region of rDNA.

**Supplementary File 2:** Differentially expressed genes (DEGs) among healthy and infected samples of (A) GC-2, (B) GC-3, (C) GC-4 and (D) GC-5.

**Supplementary File 3:** Enrichment of GO terms of molecular function for up-regulated DEGs of GC-2, (B) up-regulated DEGs of GC-3, (C) up-regulated DEGs of GC-4, (D) up-regulated DEGs of GC-5, (E) down-regulated DEGs of GC-2, (F) down-regulated DEGs of GC-3, (G) down-regulated DEGs of GC-4 and (H) down-regulated DEGs of GC-5.

**Supplementary File 4:** Enrichment of GO terms of biological process for up-regulated DEGs of GC-2, (B) up-regulated DEGs of GC-3, (C) up-regulated DEGs of GC-4, (D) up-regulated DEGs of GC-5, (E) down-regulated DEGs of GC-2, (F) down-regulated DEGs of GC-3, (G) down-regulated DEGs of GC-4 and (H) down-regulated DEGs of GC-5.

**Supplementary File 5:** Enrichment of GO terms of cellular component for up-regulated DEGs of GC-2, (B) up-regulated DEGs of GC-3, (C) up-regulated DEGs of GC-4, (D) up-regulated DEGs of GC-5, (E) down-regulated DEGs of GC-2, (F) down-regulated DEGs of GC-3, (G) down-regulated DEGs of GC-4 and (H) down-regulated DEGs of GC-5.

**Supplementary File 6:** Enrichment of KEGG pathway for up-regulated DEGs of (A) GC-2, (B) GC-3, (C) GC-4 and (D) GC-5.

**Supplementary File 7:** Enrichment of KEGG pathway for down-regulated DEGs of (A) GC-2, (B) GC-3, (C) GC-4 and (D) GC-5.

**2. Supplementary figures and tables**

**2.1. Supplementary figures captions:**

**Supplementary figure S1: Heatmap of top fifty DEGs among healthy and infected of GC-2 cultivar.**  The cut-off for the log2FC was set ≥ 2 for up-regulated DEGs and ≤ −2 for down-regulated DEGs. The cut-off for p-value was 0.05 for all DEGs. Red and blue colours indicate up-regulated and down-regulated DEGs, respectively.

**Supplementary figure S2: Heatmap of top fifty DEGs among healthy and infected of GC-3 cultivar.** The cut-off for the log2FC was set ≥ 2 for up-regulated DEGs and ≤ −2 for down-regulated DEGs. The cut-off for p-value was 0.05 for all DEGs. Red and blue colours indicate up-regulated and down-regulated DEGs, respectively.

**Supplementary figure S3: Heatmap of top fifty DEGs among healthy and infected of GC-4 cultivar.** The cut-off for the log2FC was set ≥ 2 for up-regulated DEGs and ≤ −2 for down-regulated DEGs. The cut-off for p-value was 0.05 for all DEGs. Red and blue colours indicate up-regulated and down-regulated DEGs, respectively.

**Supplementary figure S4: Heatmap of top fifty DEGs among healthy and infected of GC-5 cultivar.** The cut-off for the log2FC was set ≥ 2 for up-regulated DEGs and ≤ −2 for down-regulated DEGs. The cut-off for p-value was 0.05 for all DEGs. Red and blue colours indicate up-regulated and down-regulated DEGs, respectively.

**2.2. Supplementary tables:**

**Supplementary table S1: Details of BioSample and sequence read archive (SRA) submission of cumin transcriptome to NCBI**

| **BioProject accession No.** | **Sample name** | **Biosample accession No.** | **Study** | **Accession No.** |
| --- | --- | --- | --- | --- |
| PRJNA949548 | GC-2-H | SAMN33945322 | SRP430002 | SRR24007822 |
|  | GC-2-I | SAMN33945323 | SRP430002 | SRR24007821 |
|  | GC-3-H | SAMN33945324 | SRP430002 | SRR24007820 |
|  | GC-3-I | SAMN33945325 | SRP430002 | SRR24007819 |
|  | GC-4-H | SAMN33945326 | SRP430002 | SRR24007818 |
|  | GC-4-I | SAMN33945327 | SRP430002 | SRR24007817 |
|  | GC-5-H | SAMN33945328 | SRP430002 | SRR24007816 |
|  | GC-5-I | SAMN33945329 | SRP430002 | SRR24007815 |

**Supplementary table S2: *De novo* assembly statistics**

| **Parameter** | **Value** |
| --- | --- |
| Total trinity 'genes' | 62,845 |
| Total trinity transcripts | 65,389 |
| % GC | 44.35 |
| Contig N30 | 762 bp |
| Contig N40 | 610 bp |
| Contig N50 | 501 bp |
| Median contig length | 344 bp |
| Average contig | 456.30 |
| Total assembled bases | 29,836,958 |

**Supplementary table S3: Statistics of individual sample libraries and mapping with *de novo* assembly**

| **Plant** | **Sample** | **Total input reads** | **Mapped reads** | **% Mapping** | **Unmapped reads** | **% GC** |
| --- | --- | --- | --- | --- | --- | --- |
| Healthy | GC-2-H | 2,41,58,896 | 2,24,96,619 | 93.12 | 16,62,277 | 55 |
|  | GC-3-H | 2,23,51,066 | 2,13,26,471 | 95.42 | 10,24,595 | 56 |
|  | GC-4-H | 2,23,97,757 | 2,04,45,434 | 91.28 | 19,52,323 | 54 |
|  | GC-5-H | 1,50,40,774 | 1,36,67,897 | 90.87 | 13,72,877 | 56 |
| Infected | GC-2-I | 87,68,604 | 72,06,714 | 82.19 | 15,61,890 | 55 |
|  | GC-3-I | 3,38,00,351 | 3,13,77,204 | 92.83 | 24,23,147 | 55 |
|  | GC-4-I | 2,76,81,652 | 2,59,87,009 | 93.88 | 16,94,643 | 52 |
|  | GC-5-I | 1,57,93,177 | 1,42,22,846 | 90.06 | 15,70,331 | 56 |
| Total | | 16,99,92,277 | 15,67,30,194 | - | 1,32,62,083 | - |
| Mean | | 2,12,49,035 | 1,95,91,274 | 91.20 | 16,57,760 | 55 |
